# *Fusobacterium nucleatum*: strategies for adapting to aerobic stress

**DOI:** 10.1128/jb.00090-25

**Published:** 2025-06-06

**Authors:** Alexandra K. McGregor, Kirsten R. Wolthers

**Affiliations:** 1 Department of Chemistry, University of British Columbia, Okanagan campus 97950, Kelowna, Canada; National Institutes of Health, Bethesda, Maryland, USA

**Keywords:** *Fusobacterium nucleatum*, aerobic stress, flavodiiron proteins, glycyl-radical enzymes, methionine sulfoxide reductase, ModRS

## Abstract

*Fusobacterium nucleatum*—a gram-negative anaerobe—is commensal to the oral cavity, where it plays an important role in the maturation of the oral biofilm. The bacterium is also an opportunistic pathogen, given its association with systemic infections and cancer progression. Although residing in largely anoxic microenvironments within the oral biofilm, *F. nucleatum* encounters oxygen (O₂) present in the circulating saliva and reactive oxygen species formed endogenously, by an activated immune system or neighboring oral commensal streptococci. This review explores the bacterium’s adaptive mechanisms that enable survival under oxidative stress. We discuss how *F. nucleatum* mitigates oxidative damage and aerobic stress through common detoxifying and repair enzymes such as peroxiredoxins, methionine sulfoxide reductases, and rubrerythrin and through the activity of the recently identified multicomponent enzyme, termed butyryl-CoA oxygen oxidoreductase. Turnover by the latter enzyme enables *F. nucleatum* to exploit molecular oxygen for the conservation of energy. Additionally, we discuss how a two-component signal transduction system, ModRS, a global regulator of oxidative stress, functions in part to reprogram core metabolism to counterbalance the inactivation of a glycyl radical enzyme hypersensitive to O_2_. Our findings provide new insight into how *F. nucleatum* resists fluctuating dioxygen environments, shedding light on its persistence in extraoral sites and its potential role in disease progression.

## INTRODUCTION

*Fusobacterium nucleatum*—a gram-negative, non-spore-forming anaerobe—is commensal to the oral cavity, where it helps shape the maturation of the oral biofilm through both physical interactions and metabolic cross-feeding (1–3). Recent gene-based imaging of the human oral biofilm shows that prominent bacterial taxa form a highly structured community that creates distinct microenvironments to which individual taxa preferentially localize (4). In short, the mixed species community is radially arranged with obligate or facultative aerobes on the periphery and obligate anaerobes in the core or annulus layer. *F. nucleatum* inhabits the annulus layer adjacent to commensal streptococci localized at the periphery. Although the circulating saliva contains dissolved O_2_, aerobic metabolism by peripheral species inevitably depletes O_2_ concentration below the surface of the biofilm, such that the obligate anaerobes of the annulus and core layers reside in a largely anoxic environment (5). However, the biofilm is dynamic, and organisms residing in these anoxic micro-niches are likely subject to episodic O_2_ exposure, adversely affecting key enzymes of primary metabolism (6). As with any oral microbe, *F. nucleatum* is also subject to oxidative stress in the form of reactive oxygen species (e.g., O_2_^●−^, H_2_O_2_, ^●^OH), which can form endogenously through adventitious electron transfer to O_2_ from reduced flavin cofactors, by neighboring oral commensal streptococci or an activated host immune system (7, 8). An adaptive response to O_2_ and oxidative stress could be an important factor that enables the bacterium to play a prominent role in shaping the oral biofilm.

Tolerance to O_2_ and reactive oxygen species (ROS) could also contribute to the remarkable ability of *F. nucleatum* to disseminate to extra-oral sites, which is a major concern to human health as infection of these sites can trigger or exacerbate disease (9). For example, *F. nucleatum* has been detected in the amniotic fluid and/or fetal tissues of individuals with adverse pregnancy outcomes, including placental infections and pre-term births (10–12). The link between *F. nucleatum* and pre-term births was further supported in an animal study in which mice intravenously injected with *F. nucleatum* exhibited an increased incidence of intrauterine infections and adverse pregnancy outcomes (13).

*F. nucleatum* has been labeled an oncomicrobe due to its association with various cancers. Metagenomic studies of different cohorts show an abundance of the bacterium in adenomas and tumors of the colon, esophagus, pancreas, and breast (14–18). Critically, the abundance of *F. nucleatum* in colorectal carcinoma, esophageal, and pancreatic cancer correlates with worse patient outcomes and a higher recurrence rate (19–22). Key virulence features of *F. nucleatum* are its adhesion proteins (FadA, Fap2, and RadD), which bind to surface-exposed receptors on cancer cells, triggering signaling cascades that promote tumor growth, metastasis, and chemoresistance (23–29). The Fap2 adhesion also enables *F. nucleatum* to bind T cell immunoreceptor with Ig and ITIM domains (TIGIT), an inhibitory receptor of human natural killer cells, which inhibits this class of immune cells from killing tumors (30). The oral microbe is also highly invasive and can enter diverse cell types, including oral, colonic, placental and epithelial cells, T cells, and macrophages (31–34). Cell invasion induces a pro-inflammatory response, further exacerbating the disease. DNA linkage analysis revealed that *F. nucleatum* present in colonized tumors and amniotic fluid originated from the subgingival plaque, but it is still unclear if the organism travels hematologically, via host cells and/or the gastrointestinal system (35–37). Regardless of the route of transmission, the bacterium likely encounters O_2_ and ROS during transit, colonization of host tissue, and interaction with the host immune system. Thus, survival in the oral cavity and at systemic sites suggests that the organism has a robust system that defends against transient exposure to O_2_ and ROS.

Indeed, classical microbiological experiments have shown that *F. nucleatum* does exhibit a degree of aerotolerance, particularly in a biofilm and upon coaggregation with other bacteria (38–40). In co-culture, *F. nucleatum* supported the growth of the obligate anaerobe and oral pathogen *Porphyromonas gingivalis*, conditions in which *P. gingivalis*, in a monoculture, did not survive (41). In fact, *F. nucleatum* grew slightly but significantly better in co-culture with *P. gingivalis* under moderate oxygen levels (10%) compared with strict anaerobic conditions, indicating that it may benefit from low levels of the diatomic gas, at least in mixed cultures. The ability of *F. nucleatum* to cope with episodic oxygenation was further examined in a study that involved growing the bacteria in continuous culture under increasing partial pressures of O_2_ (42). Strikingly, cell viability was unaltered with temporary O_2_ exposure. The study also noted a shift in the fermentation end-products produced by the culture, with a depletion in butyrate coinciding with an increase in O_2_ tension. Conversely, the concentration of acetate increased with incremental increases in molecular oxygen. These results suggest that *F. nucleatum* shifts its metabolism to mitigate O_2_ exposure.

Herein, we examine environmental sources of ROS in relation to *F. nucleatum’s* natural environmental niche and its propensity to infect other tissues. Potential strategies by which *F. nucleatum* not only shields itself against episodic exposure to O_2_ but uses the diatomic gas to conserve energy are also reviewed. Similar to other anaerobes, *F. nucleatum* encodes a repertoire of enzymes that mitigate against oxidative stress by either directly detoxifying O_2_ or H_2_O_2_, repairing damaged cellular machinery, or functioning as a substitute for the damaged enzyme; each of these systems will be highlighted herein. Finally, we discuss how a two-component signal transduction system, ModRS, reprograms metabolic pathways to circumvent the O_2_ inactivation of a central metabolic enzyme.

## ENVIRONMENTAL SOURCES OF ROS

**Fig 1:**
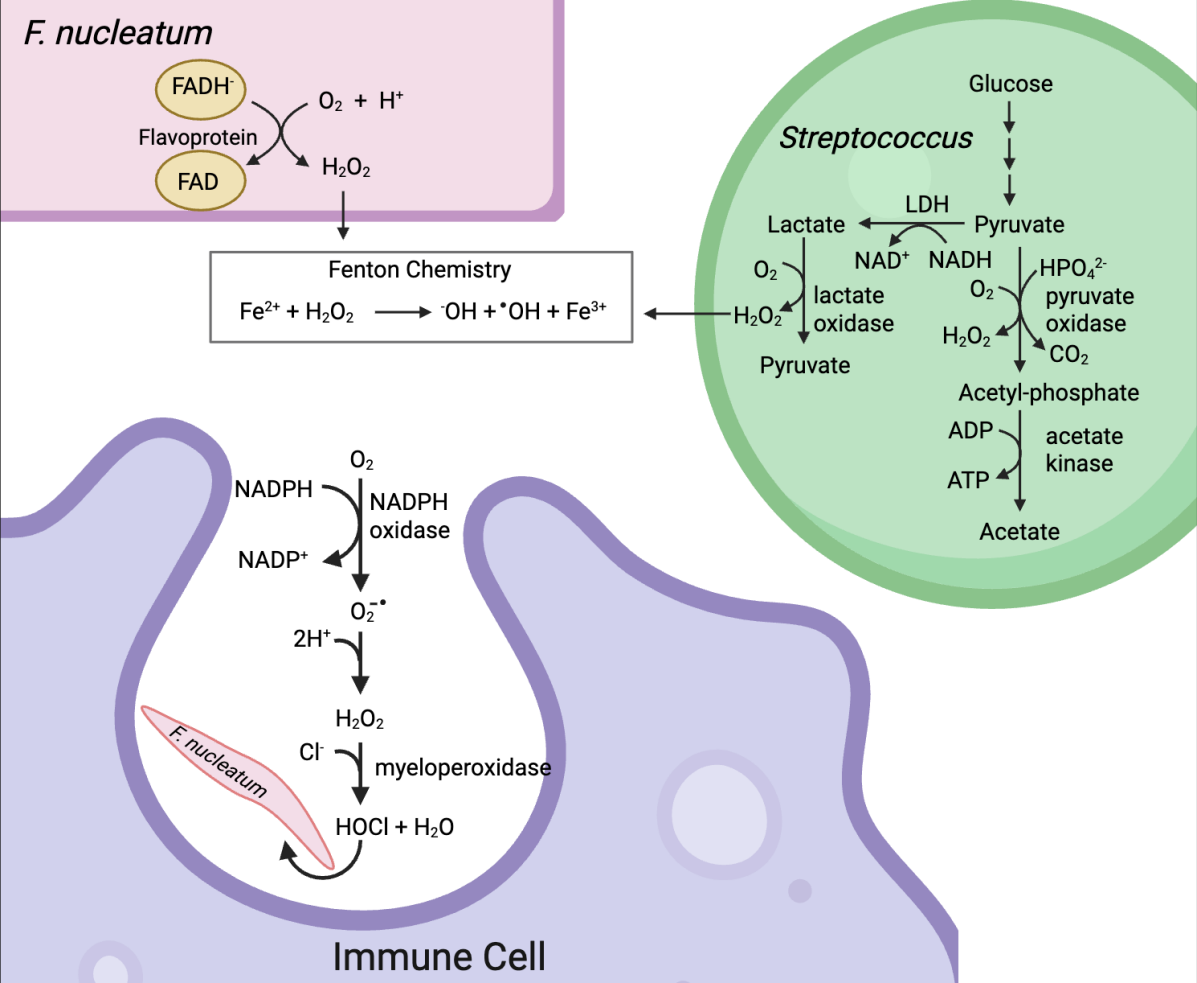
Environmental sources of ROS. *F. nucleatum* populates the oral biofilm alongside streptococci. As lactic acid bacteria, oral streptococci convert glucose to pyruvate through the EMP pathway. In the absence of O_2_, pyruvate is converted to lactate by lactate dehydrogenase, a reaction that recycles NADH to NAD^+^. In the presence of O_2_ and HPO_3_^2-^, pyruvate is oxidized to acetyl-phosphate by pyruvate oxidase, producing CO_2_ and H_2_O_2_ as byproducts. H_2_O_2_ is also a byproduct of the O_2_-dependent oxidation of lactate to pyruvate by lactate oxidase. H_2_O_2_ reacts with Fe^2+^ to form Fe^3+^, ^-^OH, and the highly potent oxidant, ^•^OH. Adventitious electron transfer from reduced endogenous flavoproteins to O_2_ is also a potential source of H_2_O_2_. Finally, activated immune cells can produce reactive oxidants, O_2_^-•^, and HOCl through the action of NADPH oxidase and myeloperoxidase.

In the annulus layer of the oral biofilm, *F. nucleatum* neighbors oral commensal streptococci (e.g., *Streptococcus sanguinis, Streptococcus gordonii, Streptococcus oralis, Streptococcus mitis*) (40). These lactic acid bacteria can grow under aerobic or anaerobic conditions and, thus, are well adapted to living in a dynamic oral biofilm with fluctuating O_2_ levels. In the absence of molecular oxygen, oral streptococci metabolize glucose via the Embden-Meyerhof-Parnas (EMP) pathway (Fig. 1). Lactic acid bacteria lack a full respiratory chain and a complete TCA cycle; consequently, pyruvate is reduced to lactate by lactate dehydrogenase, a process that regenerates NAD^+^ consumed in the EMP pathway. However, under aerobiosis, pyruvate oxidase, a flavoenzyme, converts pyruvate, orthophosphate, and O_2_ to acetyl-phosphate, CO_2_, and H_2_O_2_ (43, 44). Acetyl-phosphate is then used by acetate kinase to form ATP. Likewise, the presence of O_2_ enables lactate to be converted back to pyruvate by the action of lactate oxidase, another H_2_O_2_-generating flavoenzyme. Thus, oral commensal streptococci produce H_2_O_2_ via two flavin-dependent systems that operate during aerobiosis.

Exposure to H_2_O_2_ can damage the cell as it rapidly reacts with a labile ferrous iron, leading to the formation of a highly reactive hydroxyl radical (·OH; Fig. 1) (45). Although the hydroxyl radical can oxidize proteins and lipids, its reaction with DNA is the most damaging to the organism, since even a single lesion can be lethal or mutagenic. Production of H_2_O_2_ by oral commensal streptococci is thought to be beneficial to oral health as mixed culture studies have shown that several H_2_O_2_-producing species of oral commensal are antagonistic to the growth of *Streptococcus mutans,* a pathogenic species associated with dental caries (46–51). Until recently, it was not evident that the antagonistic effect of H_2_O_2_ extended to the oral cavity, but Kim et al. demonstrated that a high-producing H_2_O_2_ strain of *S. oralis* J22 was able to inhibit the clustering, accumulation, and spatial organization of *S. mutans* on an *ex vivo* human tooth surface. Moreover, coinfection of *S. mutans* with *S. oralis* resulted in reduced caries development in an *in vivo* rodent caries model (52). Streptococci do not possess the H_2_O_2_-degrading enzyme catalase but do encode for homologs of AhpCF, a cysteine-based peroxidase and reductase that has been shown in other bacteria to mitigate against H_2_O_2_ toxicity (53, 54). Streptococci also mitigate against the formation of ·OH by reducing their reliance on iron-containing proteins and using manganese rather than iron to metalate key enzymes (55).

Earlier work on strains of *E. coli* deficient in ROS scavenging enzymes (e.g., catalase and superoxide dismutase) revealed that H_2_O_2_ and O_2_^•-^ can also form endogenously under aerobic growth conditions as a result of adventitious electron transfer to dioxygen from flavin-dependent enzymes (45, 56, 57). For example, Hrb from *Moorella thermoacetica*, an NADPH-dependent dehydrogenase that contains an FMN cofactor and [Fe(SCys)_4_] rubredoxin domain can—at least *in vitro*—donate electrons to O_2_ instead of its natural redox partner, a flavodiiron protein (58). Likewise, acyl-CoA dehydrogenases, which possess a solvent-exposed FAD cofactor, are also noted for their ability to adventitiously donate electrons to O_2_ instead of its physiological redox partner, an electron transfer flavoprotein (59).

Cytotoxic ROS are also produced enzymatically by activated neutrophils and macrophages as a means to control the growth of extracellular or intracellular pathogens. When neutrophils engulf bacteria, they enclose them in small vesicles (phagosomes) into which superoxide is released by an activated NADPH-dependent oxidase on the internalized neutrophil membrane (Fig. 1) (60). The superoxide can rapidly dismutate to hydrogen peroxide, which myeloperoxidase uses to peroxidate chloride, forming hypochlorous acid (HOCl) (61). HOCl is membrane-permeable. It is also a strong two-electron oxidant that is very effective at both oxidation and chlorination of certain amino acids, which can lead to protein misfolding and aggregation and, ultimately, cell death (62–64).

*F. nucleatum* can be delineated into five subspecies *nucleatum*, *animalis*, *vincentii*, *fusiform,* and *polymorphum,* which are all members of the oral microbiota (65, 66). Genetic analysis of these subspecies reveals that they lack key enzymes commonly known to detoxify ROS, including superoxide dismutase, superoxide reductase, and catalase. However, they do encode two discrete enzyme systems that have been shown in other bacteria to use thiol-based chemistry to mitigate oxidative damage caused by peroxides. The first is an alkyl hydroperoxide reductase (AhpC), which belongs to a widely distributed class of peroxiredoxins that reduce hydrogen peroxide, peroxynitrite, and organic hydroperoxides (ROOH) (67). The second is methionine sulfoxide reductases (MsrA and MsrB) that reduce methionine sulfoxides that form from exposure to reactive oxidizing species (68). In addition, *F. nucleatum* encodes for rubrerythin and flavodiiron proteins demonstrated in other organisms to utilize a diiron motif to reduce H_2_O_2_ or O_2_, respectively. Herein, we review the enzymology of these repair enzymes as well as other proteins involved in the *F. nucleatum* oxidative stress response. Conservation of the genes across all subspecies of *F. nucleatum* points to their importance in adaptation to the oral biofilm and dissemination to extra-oral sites. Gene IDs are provided for *F. nucleatum* subsp. *nucleatum* ATCC 28856, the first strain to be sequenced (69). However, we have cross-listed all gene IDs with those of *F. nucleatum* subsp. *nucleatum* ATCC 23726, as the genetic trackability of this particular strain has uncovered important information on the organism’s oxidative stress response (Table S1).

## PEROXIREDOXIN AND PEROXIREDOXIN REDUCTASE

**Fig 2:**
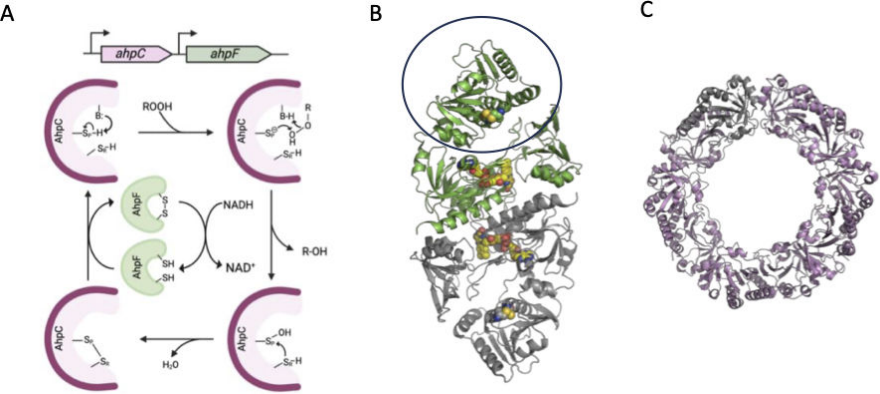
Structure and mechanism of peroxiredoxins and peroxiredoxin reductase. (**A**) The catalytic cycle of peroxiredoxin initiates with proton abstraction from the peroxidatic cysteine (-S_P_-H) resulting in a thiolate anion that attacks the alkyl peroxide or hydrogen peroxide substrate. Proton-assisted cleavage of the O-O bond results in the release of alcohol or water and the formation of a sulfenic acid (R-S-OH). A resolving cysteine (Cys–S_R_-H) then attacks the sulfenic acid intermediate, resulting in the release of water and the formation of a disulfide bond. The N-terminal domain AhpF, an NADH-dependent flavin disulfide oxidoreductase, reduces the disulfide of AhpC through a thiol exchange mechanism (67). In *F. nucleatum*, *ahpC* and *ahpF* may have independent transcriptional start sites, as RNA sequencing data suggests (74). (**B**) Molecular structure of dimeric AhpF_SE_ (PDB ID: 1HYU) with one subunit colored green and the second subunit colored gray (75). The N-terminal domain comprises two contiguous thioredoxin folds, with a single pair of redox-active cysteines (spheres) circled. In AhpF from *F. nucleatum*, this domain is located at the C-terminus. The FAD cofactor is shown as a sphere. (**C**) The decameric assembly of AhpC_SE_ is shown as a pink cartoon with one individual subunit in grey (PDB ID: 1YEX) (76).

*F. nucleatum* contains a peroxiredoxin homolog (FN1983) that shares 60% sequence identity with well-characterized peroxiredoxin of *Salmonella enterica* serovar Typhimurium (AhpC_SE_), which has been shown to confer resistance to alkyl hydroperoxides and H_2_O_2_ by reducing these compounds to alcohols and water, respectively (70–72). Based on biochemical and structural data of AhpC_SE_, the peroxide reduction initiates with a redox-active cysteine, called the peroxidate cysteine (Cys-Sp-H), attacking the peroxide substrate (Fig. 2) (67, 73). Cleavage of the O-O bond is assisted by general acid catalysis owing to the poor leaving group properties of the RO- group. This first reaction step leads to the release of alcohol (or water if the substrate is H_2_O_2_) and the formation of a sulfenic acid intermediate (Cys-S_p_-OH). A resolving cysteine (Cys–S_R_-H) then attacks the sulfenic acid intermediate, resulting in the release of water and the formation of a disulfide bond.

In *S. enterica,* a gene encoding an NADH-dependent flavin disulfide oxidoreductase (AhpF_SE_) is dicistronic with *ahpC_SE_* (77). AhpF_SE_ is a modular enzyme with C-terminal-most 314 amino acids homologous to FAD-containing thioredoxin reductase of *Escherichia coli* and the N-terminal-most 200 amino acids consisting of two fused thioredoxin folds (Fig. 2) (75, 78, 79). AhpF_SE_ catalyzes the oxidation of NADH and transfers reducing equivalents via the FAD cofactor and two conserved cysteines of the thioredoxin reductase domain (Cys345 and Cys348; AhpF_SE_ numbering) to a pair of cysteines (Cys129 and Cys132) in one of the thioredoxin folds of the N-terminus. This reduced thiol pair then reduces the disulfide bond following the turnover of AhpC_SE_ (73, 80). The gene product encoded upstream of *ahpC* (FN1984) in *F. nucleatum* is also comprised of a thioredoxin reductase domain and a second domain consisting of two fused thioredoxin folds. However, unlike AhpF_SE_, the former domain is at the C-terminus, whereas the latter is at the N-terminus. The difference in modular organization suggests that AhpF formed twice during evolution. RNA-sequence analysis performed by Ponath et al. further suggests that *ahpC* and *ahpF* each have their own promoter; thus, they are independently transcribed (74).

Peroxiredoxin was long thought to be ∼1,000 times less catalytic efficient toward H_2_O_2_ reduction than the historically better-known catalase. However, it was revealed through the development of a sensitive spectral assay in which disulfide reduction was not rate-limiting, that AhpC from *S. typhimurium* had a *k*_cat_/*K*_M_ for H_2_O_2_ of 10^7^-10^8^ M^−1^ s^−1^, surpassing that of catalase (10^6^ M^−1^ s^−1^) and approaching the limiting rate of diffusion (72, 76). Further underscoring its biological importance in scavenging H_2_O_2_ was the observation that AhpC—not catalase—was responsible for the reduction of H_2_O_2_ in *E. coli* (81). Thus, although *F. nucleatum* does not encode a catalase, it possesses homologs of AhpC and AhpF that may be sufficient to mitigate the damaging effects of alkyl hydroperoxides and H_2_O_2_.

## METHIONINE SULFOXIDE REDUCTASE

**Fig 3:**
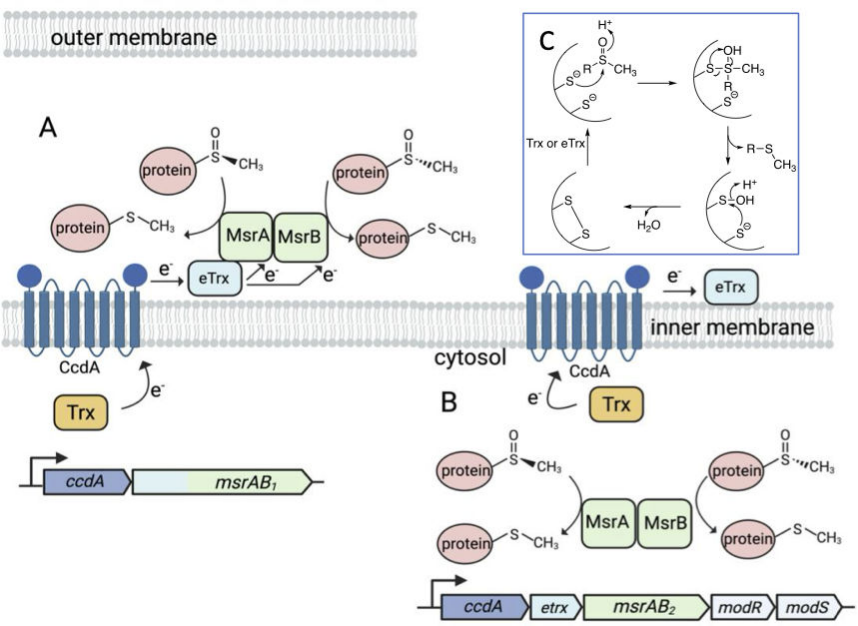
Methionine sulfoxide reductase. *F. nucleatum* contains two separate gene clusters encoding for a fused protein containing MsrA and MsrB. (**A**) At the first genetic locus (left), *msrAB_1_* encodes MsrAB_1_ with an N-terminal thioredoxin domain, similar to PilB from *N. meningitidis* (96, 97). Contiguous with *msrAB_1_* is *ccdA*, encoding CcdA, a transmembrane protein that likely shuttles electrons from intracellular Trx to MsrAB_1_ for the reduction of methionine sulfoxides (97). (**B**) At the second locus (right), *msrAB_2_* is clustered with *ccdA*, *eTrx modR,* and *modS,* which encode CcdA, eTrx (extracellular thioredoxin), and ModRS, a two-component signal transduction system that acts as a global oxidative stress response regulator (98). (**C**) The proposed Msr reaction begins with a nucleophilic attack on the sulfoxide by a deprotonated active site cysteine, forming a tetrahedral transition state that undergoes rearrangement. This leads to the release of methionine and the formation of a sulfenic acid intermediate (94). A second deprotonated cysteine then attacks the sulfenic acid, resulting in the formation of a disulfide bond and the release of water. The active MsrAB enzyme is regenerated through a disulfide exchange mechanism involving a thioredoxin-like protein (95).

The thioether side chain of methionine is particularly susceptible to oxidation by ROS and reactive chlorine species, leading to protein dysfunction (82). Of potential oxidants, ^•^OH and HOCl are the most potent oxidizers of methionine, exhibiting second-order rate constants of 3.8 × 10^7^ M^−1^ s^−1^ and 8.9 × 10^9^ M^−1^ s^−1^ (pH 7), respectively (83, 84). By comparison, the corresponding rate constant for H_2_O_2_ oxidation of methionine is 6 × 10^−3^ M^−1^ s^−1^ (85)! Oxidation of the thioether side chain generates methionine sulfoxide (-SO-) in either the (R-) or (S-) diastereomeric form. Methionine sulfoxide reductases (Msr), which are found in most living organisms, repair the damaged side chain by catalyzing the reduction of methionine sulfoxide to methionine, a reaction that consumes two protons and generates a water molecule (86). Many organisms encode MrsA and MsrB, which exhibit stereospecificity toward the (R-) or (S-) diastereomers of methionine sulfoxide, respectively (87–89). In *F. nucleatum*, as in some other bacteria, the two reductases are translated as a fusion protein labeled MsrAB. Although MsrA and MsrB are structurally distinct, they both use a thiol-dependent mechanism to reduce the sulfoxide (90–93). The proposed reaction initiates with nucleophilic attack of the sulfoxide by a deprotonated active site cysteine, which generates a tetrahedral transition state that undergoes rearrangement, resulting in the release of methionine and formation of a sulfenic acid intermediate (Fig. 3) (94). The attack of the sulfenic acid intermediate by a second deprotonated cysteine results in the formation of a disulfide bond and the release of water. Regeneration of an active MsrAB occurs through a disulfide exchange mechanism involving a thioredoxin-like protein (95).

*F. nucleatum* contains two copies of *msrAB*, *msrAB_1_* (FN0188), and *msrAB_2_* (FN0803), encoded at distinct gene clusters (Fig. 3). MsrAB_1_ (473 amino acids) contains an additional N-terminal thioredoxin-like domain fused to the tandem Msr A and B components. This domain organization is similar to that of PilB from *Neisseria gonorrhoeae* (50% sequence identity) (92). Biochemical studies of PilB confirmed that the N-terminal thioredoxin domain reduces the disulfide bridge formed during the turnover of MsrAB (96). Notably, homologs of PilB have a narrow phylogenetic distribution, being found to date in select genera within the families of *Neisseriaceae* (*Eikenella* sp., *Neisseria* sp.*,* and *Kingella* sp.), *Moraxellaceae* (e.g., *Moraxella* sp. and *Psychrobacter* sp.), and *Fusobacteriaceae*.

In *F. nucleatum*, *msrAB_1_* is dicistronic with a gene encoding a cytochrome *c* biogenesis A (CcdA)-like protein. NMR experiments of an archaeal CcdA homolog revealed that this transmembrane protein employs a single pair of cysteine residues and a conformational switch mechanism that enables the transfer of reducing equivalents derived from cytoplasmic thiols (e.g., reduced thioredoxin) to extracellular disulfides (e.g., oxidized periplasmic thioredoxins) (97, 99, 100). *F. nucleatum* does encode thioredoxin (*trx*; FN0093) and an NADPH-dependent thioredoxin reductase (*trxR*; FN1163) that may supply reducing equivalents to CcdA. Analysis of MsrAB_1_ by SignalP 6.0 indicates a periplasmic targeting sequence, suggesting that MsrAB_1_, along with the accessory proteins, protects periplasmic and outer membrane proteins from exogenous oxidative stress (101).

MsrAB_2_ (FN0188; 298 amino acids) is part of a five-gene cluster that also includes genes for a two-component signal transduction system (ModRS), a thioredoxin-like protein (eTrx), and a cytochrome *c* (CcdA)-like protein. Notably, these five genes were upregulated upon exposure of *F. nucleatum* to 1 mM H_2_O_2_. Although this concentration of H_2_O_2_ is not physiologically relevant, it may have resulted in sufficient protein damage to trigger the upregulation of *msrAB_2_*. The importance of MsrAB_2_ in the oxidative stress response was established through a Δ*msrAB_2_* strain, which exhibited higher sensitivity to H_2_O_2_ and a weakened ability to invade colorectal epithelial cells and survive in macrophages compared to the parental strain (98). These resulting phenotypes mirror those of Δ*msrAB* strains of other bacterial pathogens, which also exhibited reduced fitness and virulence in different infection models (102–106). Unlike MsrAB_1_, analysis of the MsrAB_2_ (FN0188) sequence using SignalP 6.0 [14] revealed that the protein does not carry a known signal for translocation to the periplasm. Thus, MsrAB_2_ is likely cytosolic and functions to reduce methionine sulfoxides on intracellular proteins. However, the adjacent thioredoxin-like gene does encode for an N-terminal signal sequence, and the presence of *ccdA* in the operon suggests that the combined function of CcdA and eTrx is to translocate electrons from reduced thiol proteins in the cytosol to a periplasmic eTrx for reduction of oxidatively damaged proteins.

## RUBRERYTHRINS

**Fig 4:**
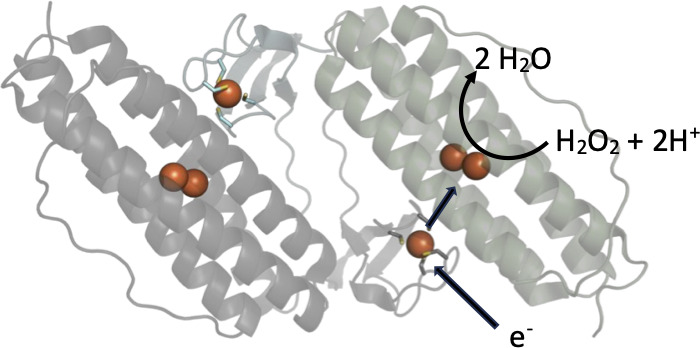
Structure of rubrerythrin. Head-to-tail dimeric structure of rubrerythrin from *D. vulgaris* (PDB ID 1LKM) (115). The C-terminal rubredoxin domain with the [Fe(SCys)_4_] domain of one monomer is colored cyan while the N-terminal four ⍺-helical bundle of the ferritin family is colored light green. The second monomer is colored gray. The iron atoms are depicted as orange spheres. The [Fe(SCys)_4_]^2+^ redox cluster receives electrons (one at a time) from an external reductant, which it subsequently transfers to the diiron motif present in the ⍺-helical bundle rubredoxin domain. Two sequential one-electron transfers facilitate the peroxidase activity of rubrerythrin.

Rubrerythrin is a non-heme iron protein first isolated from the anaerobic sulfate-reducing bacterium *Desulfovibrio vulgaris* (107). Extensive genetic and microbiological studies of rubreythrins from *D. vulgaris* and other anaerobes show that this enzyme mitigates against oxidative stress primarily by reducing hydrogen peroxide to two equivalents of H_2_O (108–111). *In vitro*, rubrerythrin from *Clostridium difficile* and *Clostridium acetobutylicum* was also shown to function as an O_2_ reductase, but the measured activity was 2 to 7-fold lower compared with peroxidase activity (112, 113). Rubrerythrins comprise an N-terminal four ⍺-helical bundle of the ferritin superfamily and a C-terminal rubredoxin domain (Fig. 4) (114, 115). The former domain provides residues for the assembly of a diiron motif. In contrast, the latter domain contains a [Fe(SCys)_4_] site, whereby a single iron atom is coordinated via sulfur atoms of four cysteine residues. The reduced diiron site reacts directly with hydrogen peroxide, whereas the [Fe(SCys)_4_] cluster transfers electrons from an exogenous donor to a μ−1,2-H_2_O_2_ intermediate that forms at the diiron site (116). *In vitro* studies have established that rubreythrin from *C. acetobutylicum* and *C. difficile* can receive electrons from an NADH-dependent rubredoxin oxidoreductase and a rubredoxin (112, 113). *F. nucleatum* encodes for rubreythrin (FN0455), but the role of this protein in the oxidative stress response has not been determined.

## BUTYRYL-COA OXYGEN OXIDOREDUCTASE

Flavodiiron proteins (FDPs) are commonly found in obligate anaerobes, where they mitigate oxidative and/or nitrosative stress by reducing O_2_ to H_2_O and/or NO to N_2_O (117–119). The simplest FDPs (referred to as class A FDPs) comprise two domains: an N-terminal metallo-β-lactamase module harboring a catalytic diiron center and a C-terminal flavodoxin module that binds FMN (120). This core unit assembles into a “head” to “tail” homodimer, enabling efficient electron transfer from the FMN of one monomer to the diiron center of the second monomer (121, 122). Bioinformatic analysis has revealed that FDPs can be fused to additional domains (e.g., rubredoxin domain, a domain with a [4Fe-4S] cluster, an NAD(P)H:flavin oxidoreductase, and NADH: rubredoxin oxidoreductase), which all ostensibly serve to shuttle electrons to the FDP (123, 124). The variations in domain assemblies have resulted in nine distinct classes of FDP (class A to I). For most FDPs studied to date, a reduced pyridine nucleotide serves as the primary source of electrons for reducing O_2_ and/or NO at the diiron catalytic site (120, 125–127).

*F. nucleatum* encodes for two FDPs: FN1423 and FN0512. The latter is monocistronic, whereas the former is of particular interest, as it is bicistronic with a gene encoding a multidomain enzyme comprising an N-terminal butyryl-CoA dehydrogenase (Bcd) domain, the C-terminus of the ⍺-subunit of electron transfer flavoprotein (EtfA), and a rubredoxin (128). We have termed this fusion protein butyryl-CoA reductase (BCR). Our biochemical analysis of BCR and the adjacently encoded FDP revealed that the two proteins form an ⍺_4_β_4_ complex and together couple the oxidation of butyryl-CoA to crotonyl-CoA with the reduction of O_2_ to H_2_O. Unlike other FDPs, the BCR-FDP complex did not exhibit NO reductase activity; thus, it functions solely as a butyryl-CoA oxygen oxidoreductase (BOOR), which the enzyme now labeled (128). Using butyryl-CoA, a relatively weak thermodynamic reductant (*E*°’ = −10 mV), instead of the more moderate reductant, NAD(P)H (*E*°’ = −320 mV) to reduce O_2_ to H_2_O (*E*°’ = +820 mV) enables *F. nucleatum* to conserve free energy in the detoxification of O_2_ (129). Notably, differential gene expression analysis revealed that the BCR gene was upregulated during planktonic growth compared to biofilm growth, environmental conditions where the bacteria may be more likely exposed to O_2_ from the surrounding environment (130).

**Fig 5:**
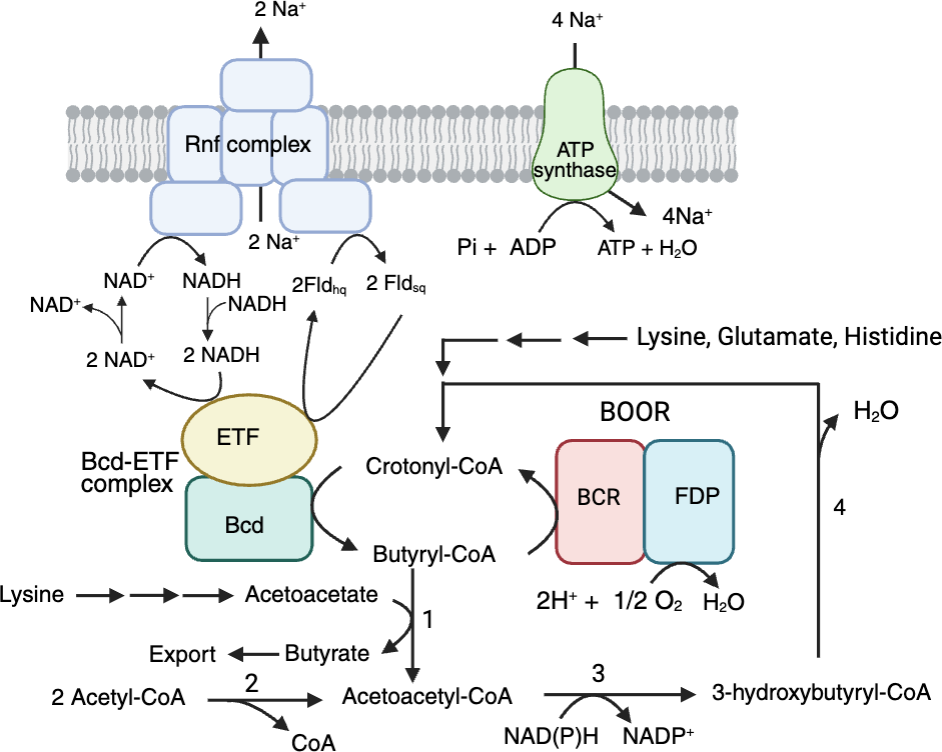
Energy conservation by butyryl-CoA oxygen-oxidoreductase (BOOR). The butyryl-CoA reductase (BCR) subunit of BOOR catalyzes the oxidation of butyryl-CoA to crotonyl-CoA, transferring reducing equivalents to the flavodiiron protein (FDP) subunit, enabling the reduction of O_2_ to H_2_O at the diiron motif (128). Crotonyl-CoA produced by BOOR turnover can be used by the bifurcating Bcd-ETF complex to generate 2-electron reduced flavodoxin (Fld_hq_) at the expense of NADH oxidation. Fld_hq_ can then transfer electrons to the Rnf complex, regenerating the one-electron reduced semiquinone form of the flavodoxin (Fld_sq_) (139, 140). This electron-transfer step is used to reduce NAD^+^ to NADH and create an ion motive force across the cytoplasmic membrane, which then can be used for ATP synthesis by an ATP synthetase. Acetoacetate:butyryl-CoA transferase (FN0272 and FN0273, labeled 1) can transfer the CoA to acetoacetate (formed during lysine fermentation) to form butyrate and acetoacetyl-CoA. This product can also be formed by acetyl-CoA acetyltransferase (FN0495, labeled 2) with two equivalents of acetyl-CoA. Acetoacetyl-CoA then undergoes reduction by 3-hydroxybutyryl-CoA dehydrogenase (FN1020) to 3-hydroxybutyryl-CoA, which condenses to crotonyl-CoA by hydroxybutyryl-CoA dehydratase (FN1019).

BOOR conversion of butyryl-CoA to crotonyl-CoA is another mechanism by which *F. nucleatum* conserves energy. *F. nucleatum* preferentially utilizes lysine, histidine, and glutamate as a source of carbon and nitrogen, and the product of these three amino acid fermentation pathways is crotonyl-CoA (Fig. 5) (131–134). Crotonyl-CoA serves as the terminal electron acceptor for the bifurcating butyryl-CoA dehydrogenase-electron transfer flavoprotein (Bcd-ETF; FN0783-FN0785) complex. Bcd-Etf, first characterized from the strict anaerobe, *Clostridium kluyveri,* couples NADH-dependent reduction of crotonyl-CoA (an exergonic reaction) to butyryl-CoA with the NADH-dependent reduction of flavodoxin (an endergonic reaction) (135, 136). The reduced flavodoxin then transfers electrons to the Rnf (*Rhodobacter capsulatus n*itrogen *f*ixation) complex, which facilitates the reduction of NAD^+^ and the establishment of the Na^+^-electrochemical gradient (137, 138). The ion motif force can then be used to drive ATP synthesis or import amino acids. Genetic disruption of a component of the Rnf complex (*rnfC*) in *F. nucleatum* underscored the importance of this respiratory enzyme (139). The deletion strain exhibited reduced amino acid fermentation and ATP production, altered cell morphology and growth, and reduced biofilm formation and virulence. The Bcd-Etf and Rnf complexes are key energy-conserving and converting systems for the fermentative metabolism of *F. nucleatum*; BOOR integrates into this respiratory system in the presence of O_2_. The ability of *F. nucleatum* via BOOR to exploit O_2_ for energy conservation may account for the modest increase in bacterial growth observed in 10% O_2_ compared with strict anaerobic conditions, at least in mixed cultures with *P. gingivalis* (40).

BOOR turnover may also account for reduced concentrations of butyrate in the media that resulted from temporary exposure of the bacterial culture to O_2_ (42). Butyrate is released from butyryl-CoA through the action of acetoacetate:butyryl-CoA transferase (FN0272, FN0273), which relocates the CoA moiety to acetoacetate (product of lysine fermentation). The resulting acetoacetyl-CoA undergoes reduction to 3-hydroxybutyryl-CoA by 3-hydroxybutyryl-CoA dehydrogenase (FN1020), and hydroxybutyryl-CoA dehydratase (FN1019) condenses 3-hydroxybutyryl-CoA to crotonyl-CoA. BOOR turnover redirects butyryl-CoA to crotonyl-CoA, resulting in less butyrate formation. Thus, O_2_ appears to modulate the amount of butyrate exported by the bacterium, which could impact oral health, given the multifaceted role of the short-chain fatty acid in modulating the host immune system and the growth of other oral microbes (141).

From another perspective, the combined action of BOOR, Rnf, and Bcd-ETF may contribute to the aerotolerance of *F. nucleatum* and its ability to colonize a wide array of extra-oral tissues. Electron bifurcation by Bcd-Etf regenerates butyryl-CoA (fueling further BOOR turnover) while producing low-potential reductants (e.g., reduced flavodoxin) at the expense of NADH. The Rnf complex integrates into the oxidative stress response by regenerating NADH and oxidized flavodoxin for further butyryl-CoA production by Bcd-Etf. It is worth noting that this system is not unique to *F. nucleatum*, as many obligate anaerobes of the *Firmicute* phyla, including dominant butyrogenic bacteria that are beneficial to gut health, encode for BOOR, Rnf, and Bcd-Etf (128, 142). The BOOR/Rnf/Bcd-Etf also mimics, in part, the pyruvate oxidase and lactate oxidase system of lactic acid bacteria, which also exploit O_2_ to enhance ATP yield (44).

## METABOLIC REPROGRAMMING BY MODRS

As stated above, the two-component signal transduction system (ModRS) was shown to upregulate a number of gene clusters involved in methionine sulfoxide reduction (*msrAB_2_*) as well as gene loci involved in the catabolism of histidine, methionine, and ethanolamine (98). Genes downregulated include those involved in *de novo* purine biosynthesis and the phosphotransferase system for fructose utilization. We suggest that this suite of genes not only compensates for the damaging effects of ROS but also counteracts the detrimental effects of O_2_. Even transient exposure to O_2_ leads to the endogenous formation of ROS, as the oral microbe encodes a flavin-dependent dehydrogenase and reduced flavodoxins that can *in vitro* spuriously donate electrons to O_2_ when populating the reduced hydroquinone state (unpublished data). Given that the damaging effects of ROS are a consequence of O_2_ exposure, ModRS may function to circumvent the damaging effects of both.

**Fig 6:**
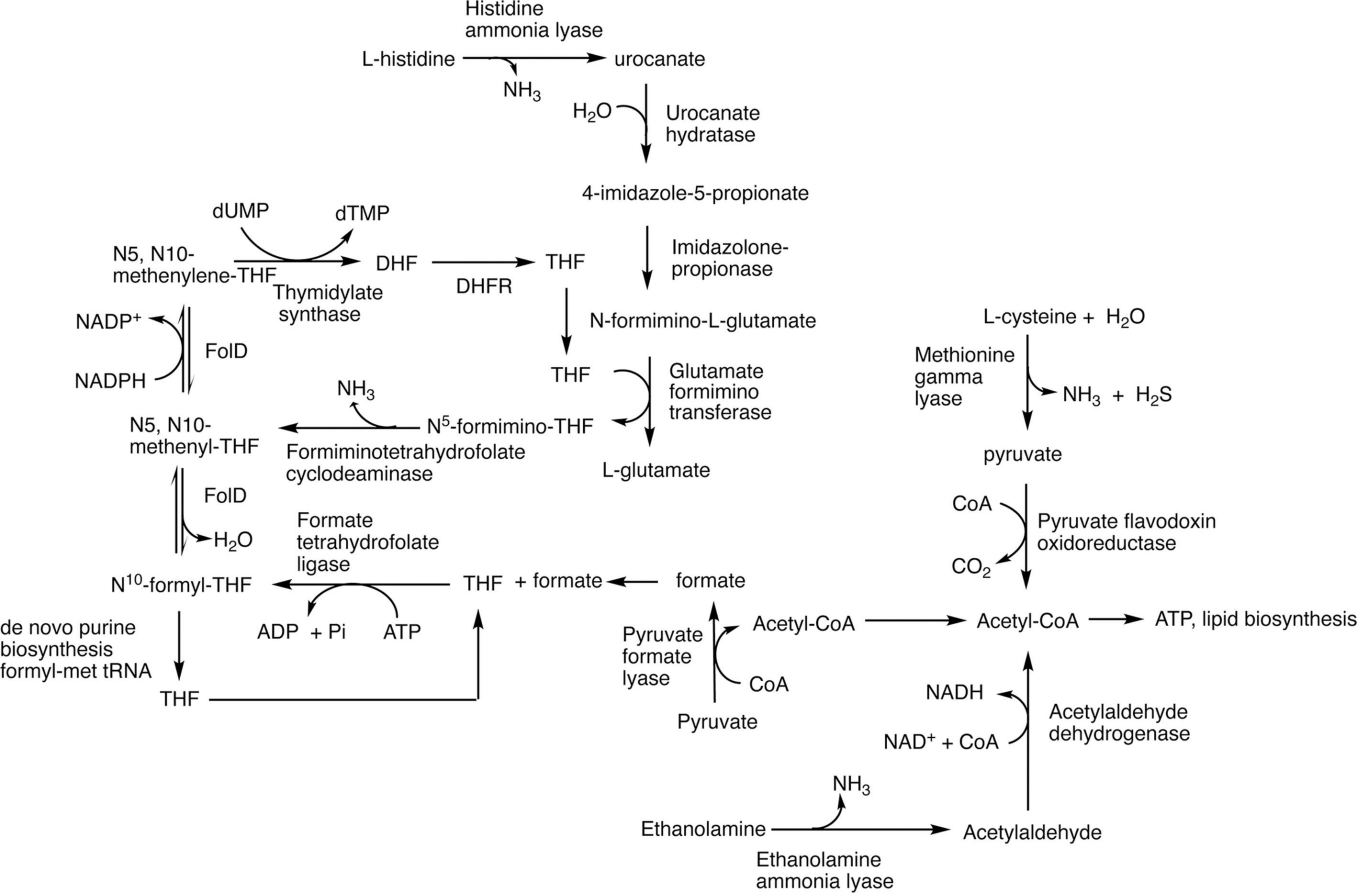
Circumvention O_2_ inactivation of pyruvate formate lyase. Molecular oxygen irreversibly inactivates pyruvate formate lyase, which converts CoA and pyruvate to formate and acetyl-CoA. Formate can be used by formate tetrahydrofolate ligase to form N^10^-formyl-tetrahydrofolate (N^10^-formyl-THF), which feeds into the one-carbon folate pool required for *de novo* purine, thymidylate, and formyl-met tRNA biosynthesis. FolD is a bifunctional tetrahydrofolate dehydrogenase and cyclohydrolase that interconverts N^10^-formyl-THF, to N^5^, N^10^-methenyl-THF to N^5^, N^10^-methenylene-THF. Following the transfer of the methyl group to dUMP by thymidylate synthase, the resulting dihydrofolate (DHF) is converted to THF by dihydrofolate reductase. ModRS upregulates histidine fermentation, which can contribute to the one-carbon folate pool as shown. Likewise, ModRS enhances the expression of enzymes and proteins involved in the conversion of ethanolamine to acetyl-CoA and L-cysteine to acetyl-CoA (98).

The upregulation of histidine catabolism helps support one-carbon folate metabolism following the O_2_-inactivation of a glycyl-radical enzyme, pyruvate formate lyase. Like other bacteria, *F. nucleatum* degrades histidine to glutamate and ammonia via the histidine utilization (hut) system (Fig. 6) (134, 143). The first three steps of the pathway are universal and initiated with histidine ammonia lyase (encoded by *hutH,* FN1406), catalyzing the non-oxidative deamination of L-histidine to *trans*-urocanic acid and ammonia. In the second step, urocanase (imidazolone-propionate hydrolase; *hutU*) employs a tightly bound NAD^+^ cofactor for the conversion of *trans*-urocanic acid to hydroxy-imidazolyl-propionate, which tautomerizes spontaneously to form 4-imidazolone-propionate. Imidazolonepropionase (encoded by *hutI;* FN1404) catalyzes the third step: hydrolysis of the carbon-nitrogen bond in 4-imidazolone-5-propionate to yield *N*-formimino-L-glutamate.

Further degradation of *N*-formimino-L-glutamate can occur via one of three pathways; it can be hydrolyzed to glutamate and formamide by formimino-L-glutamate hydrolase (*hutG*) or deaminated by iminohydrolase (*hutF*) to form ammonia and formylglutamate (*hutF*), which is then converted to glutamate and formate by formylglutamate deformylase. In a third pathway, which occurs in mammals and some bacteria, including *F. nucleatum,* degradation of *N*-formimino-L-glutamate is coupled to one-carbon folate metabolism (144). Glutamate formiminotransferase (FN1407) initially transfers the formino group from *N*-formimino-L-glutamate to the N5 of tetrahydrofolate (THF) to form N^5^-formimino-THF and glutamate. Formiminotetrahydrofolate cyclodeaminase (FN1405) then catalyzes the deamination of N^5^-forming-THF to form ammonia and N^5^, N^10^-methyl-THF. The latter product can be directly converted to N^10^-formyl-THF or N^5^, N^10^-methylene-THF by the bifunctional enzyme (FN1488) methylenetetrahydrofolate dehydrogenase /methenyltetrahydrofolate cyclohydrolase. In *F. nucleatum*, N^5^, N^10^-methylene-THF is an essential cofactor for thymidylate biosynthesis. At the same time, N^10^-formyl-THF is used in two enzymatic steps for the *de novo* biosynthesis of purines and formylation of the initiator tRNA. All of the above-mentioned genes for L-histidine catabolism are clustered along with genes that encode an aminoacyl-histidine dipeptidase, histidine permease, and a membrane-spanning protein with an unknown function.

**Fig 7:**
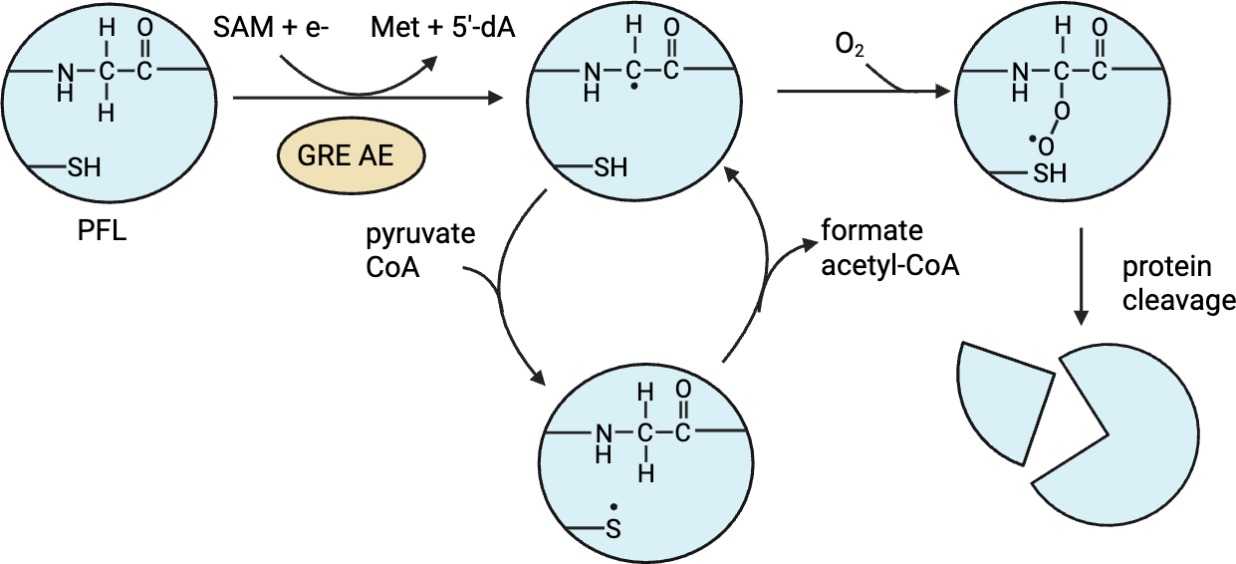
Activation and O_2_ inactivation of pyruvate formate lyase (PFL). PFL is activated by a glycyl radical enzyme activating enzyme (GRE-AE) which uses *S*-adenosyl-L-methionine (SAM), an electron derived from a reduced flavodoxin, and an embedded [4Fe-4S] redox center to form a highly reactive 5ʹ-deoxyadenosyl radical, which then abstracts hydrogen from an active site glycine to form a glycyl radical on PFL, forming the products, methionine and 5ʹ-deoxyadenosine (5ʹ-dA) (145, 146). The glycyl radical then abstracts hydrogen from an active site cysteine, which enables conversion of pyruvate and CoA to acetyl-CoA and formate. Molecular oxygen reacts with the glycyl radical to form a peroxyl radical, which leads to the cleavage of the polypeptide backbone at the glycyl radical, rendering the protein inactive (156).

Notably, *F. nucleatum* can also form N^10^-formyl-THF by formate tetrahydrofolate ligase (FN2082), which uses ATP to add formate to THF. This latter reaction may be impeded in the presence of O_2_, as formate is generated by pyruvate formate lyase, a member of the glycyl radical enzyme family, which is known for its hypersensitivity to O_2_ (145). PFL–a central player in anaerobic primary metabolism reversibly converts pyruvate and coenzyme A to formate and acetyl-CoA (Fig. 7). PFL employs a radical-based mechanism for this reaction to circumvent the bonding electrons’ natural tendency to remain with the C2 during the cleavage of the C1-C2 atoms of pyruvate. The resting form of PFL contains a glycyl radical that is formed through the action of a glycyl-radical enzyme activating enzyme (GRE-AE), a member of the *S*-adenosyl-methionine-dependent superfamily of enzymes (145–147). GRE-AE utilizes a [4Fe-4S]^1+^ cluster, which functions to reductively cleave S-adenosyl-L-methionine to form a 5'-deoxyadenosyl radical that abstracts hydrogen from an active site glycine to generate a glycyl radical (148–151). To initiate catalysis, the glycyl radical is proposed to abstract hydrogen from an active site cysteine to form a transient thiyl radical, which then abstracts hydrogen from pyruvate, enabling radical-based cleavage of the C1-C2 bond (152, 153). Following product formation, the glycyl radical is regenerated for the next round of catalysis (145). In the absence of O_2_, the glycyl radical is stable due to a captodative effect that arises from the combination of electron donation and withdrawal from the neighboring amine and carbonyl groups, respectively (154). In fact, under strict anaerobic conditions, this organic radical can persist for several days *in vitro* and catalyze numerous turnovers following the initial activation step by the GRE-AE (155). However, the glycyl radical can rapidly convert to peroxide radical in the presence of O_2_, which leads to cleavage of the polypeptide backbone at the site of the glycine, resulting in irreversible enzyme inactivation (156, 157). In order to cope with the physical loss of a part of the protein, some facultative anaerobes express small proteins called autonomous glycyl radical cofactors that have high sequence similarity to the cleaved region of the glycyl radical enzyme (158). These autonomous glycyl radical cofactors replace the cleaved portion of the enzyme, restoring enzymatic activity (159). However, *F. nucleatum* does not contain an autonomous glycyl radical cofactor homolog. Instead, it partially copes with irreversible PFL damage by upregulating histidine catabolism to maintain one-carbon folate metabolism. ModRS-mediated down-regulation of *de novo* purine biosynthesis, which utilizes N^10^-formyl THF in two steps, may also be a means to spare one-carbon folate metabolism for thymine and protein synthesis.

The upregulation of genes involved in the transport and catabolism of ethanolamine and methionine would ostensibly bolster the intracellular concentration of the central metabolite acetyl-CoA impacted by a loss of PFL activity. Ethanolamine is a component of the membrane lipid, phosphatidylethanolamine present in eukaryotes and prokaryotes, and the ability to catabolize ethanolamine is facilitated through the ethanolamine utilization (*eut*) gene cluster. Ethanolamine ammonia lyase—encoded by *eutB* and *eutC*—catalyzes the first step of the reaction, the radical-based deamination of ethanolamine to form acetaldehyde and ammonia (160). Acetaldehyde dehydrogenase, encoded by *eutE,* subsequently converts acetaldehyde and coenzyme A to acetyl CoA.

ModRS also upregulates the gene for methionine ɣ-lyase (*megl* FN1419) and the nearby gene (FN1421) encoding for a pyruvate flavodoxin oxidoreductase. Although methionine γ-lyase catalyzes the ⍺ɣ-elimination of methionine to form methyl mercaptan, NH_3_, and 2-isobutyrate, it also can catalyze the ⍺β-elimination of L-cysteine to form H_2_S, pyruvate, and ammonia (161). In fact, of the four pyridoxal 5'-phosphate-dependent enzymes known to catalyze the desulfurization of L-cysteine, methionine γ-lyase exhibited the highest intracellular activity (162). The pyruvate formed from methionine γ-lyase can serve as a substrate for pyruvate flavodoxin oxidoreductase, which catalyzes the oxidative decarboxylation of pyruvate, transferring electrons to flavodoxin and the acetyl moiety to CoA to form acetyl-CoA. Thus, the combined action of methionine γ-lyase and pyruvate flavodoxin oxidoreductase can supplement the acetyl-CoA pool.

## RIBONUCLEOTIDE REDUCTASES

**Fig 8:**
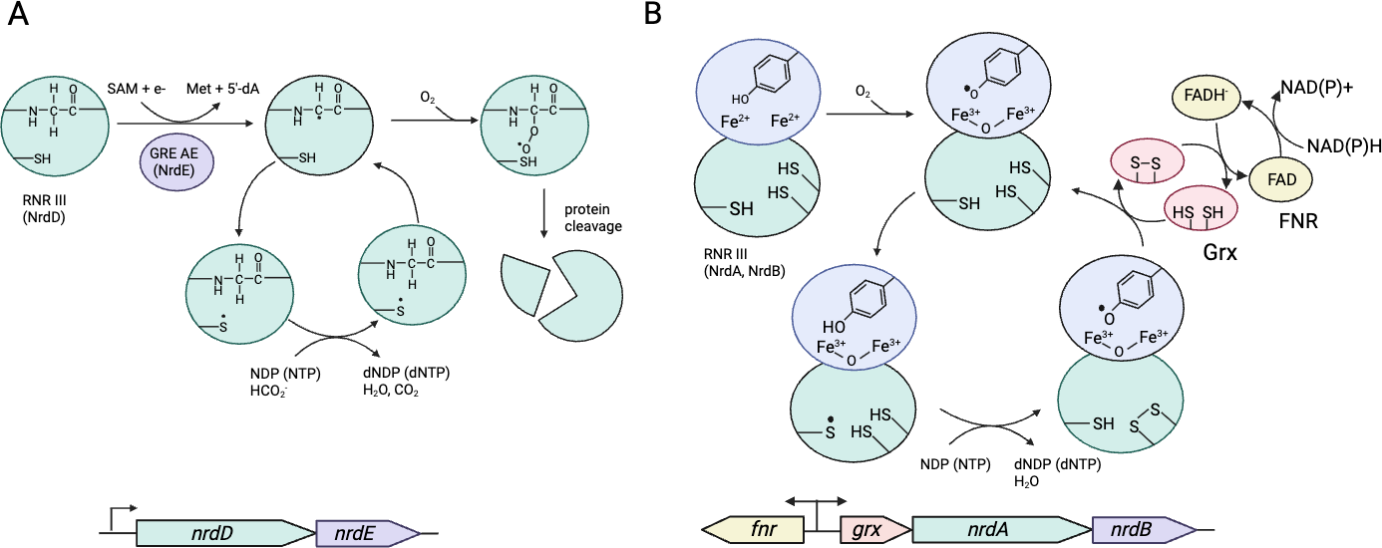
Class I and class III ribonucleotide reductases. (**A**) The class III ribonucleotide reductases (RNRs) are glycyl radical enzymes encoded by *nrdD*. The glycyl radical is installed by a glycyl radical enzyme activating enzyme (GRE-AE) encoded by *nrdE*. The GRE-AE reductively cleaves S-adenosyl-L-methionine to form methionine and a 5'-deoxyadenosyl-radical. The latter abstracts hydrogen from an active site glycine residue of NrdD. The glycyl radical then abstracts hydrogen from a cysteine to form a thiyl radical, which initiates the conversion of nucleotides to deoxynucleotides. Reducing equivalents for this reaction are supplied by formate, forming CO_2_ as a byproduct. As with pyruvate formate lyase, O_2_ reacts with the glycyl radical resulting in cleavage of the polypeptide backbone. (**B**) Class Ia RNR contains a diferrous iron site on the NrdB (R2) subunit, which reacts with O_2_ to form a diferric oxygen center and a stable tyrosyl radical. The radical is transmitted to a cysteine in the NrdA (R1) subunit to form a thiyl radical that facilitates the conversion of nucleotides to deoxynucleotides. The reducing equivalents for the reaction are supplied by a pair of thiols for two cysteine side chains. The resulting disulfide is likely reduced by a glutaredoxin (Grx), encoded by *grx* and pyridine-nucleotide dependent ferredoxin-like flavin reductase (FNR), transcribed in the reverse direction (*fnr*).

Ribonucleotide reductases (RNR) are essential for providing a balanced pool of deoxynucleotides required for DNA synthesis and repair (163, 164). There are three structurally distinct classes of RNR (I, II, and III), differentiated in part by their use of different cofactors to generate a thiyl radical required to reduce the ribose ring (Fig. 8) (152, 165). Class I RNR generates the thiyl radical by incorporating O_2_ into a binuclear metal cluster (Fe or Mn). This triggers the formation of a stable tyrosyl radical, which, through a long-range proton-coupled electron transfer, forms thiyl radical (166). Critically, O_2_ is not required for every equivalent of dNTP product, as the tyrosyl radical is regenerated after each catalytic cycle (167). In contrast, radical formation in Class II and III enzymes is independent of O_2_. Class II uses adenosylcobalamin (coenzyme B12) to generate a 5'-deoxyadenosyl radical, which then abstracts hydrogen from the active site cysteine to form a thiyl radical. Finally, Class III RNR (NrdD) first forms a glycyl-radical—generated by a GRE-AE (NrdG) using radical *S*-adenosylmethionine (SAM) [4Fe4S]^1+^ chemistry—for radical propagation to the active site cysteine. Class II RNR tolerates O_2_, but NrdD, being a member of the glycyl-radical enzyme family, irreversibly inactivates in the presence of O_2_. Consequently, NrdD and its associated GRE-AE (NrdG) are found in anaerobic bacteria, including *F. nucleatum* (FN0311 and FN0312).

Notably, *F. nucleatum* also encodes for a Class I RNR (FN0102-FN0103, NdrAB), which is unexpected given the reliance of Class I RNR on O_2_ for the formation of the tyrosyl radical. Presumably, *F. nucleatum* utilizes NdrAB to maintain a balanced dNTP pool in the event of O_2_ inactivation of NrdD, exploiting temporary aerobiosis to generate the tyrosyl radical of NdrAB. The occurrence of Class I and III RNR in an anaerobe is not unprecedented, as *Bacteroides fragilis*, an obligate anaerobe capable of long-term survival in the presence of air, also encodes Class I and Class III RNR, with the former being induced during oxidative stress (168). However, it is perplexing as to why *F. nucleatum* did not employ a class II RNR to supplant the role of an inactive NrdD, given that class II RNR is O_2_ tolerant and that *F. nucleatum*—unlike *Bacteroides fragilis*—biosynthesizes adenosylcobalamin, the radical initiator for Class II RNR (169). Regardless, the presence of a Class I RNR constitutes an additional layer of defense against O_2_ and another system by which *F. nucleatum* can exploit O_2_ for survival.

## CONCLUDING REMARKS

In summary, *F. nucleatum* can adapt to fluctuating oxygen environments through a diverse arsenal of oxidative stress mitigation strategies. These include enzymatic detoxification via peroxiredoxins, methionine sulfoxide reductases, rubrerythrin, and flavodiiron proteins, as well as metabolic shifts that minimize ROS-induced damage. Moreover, *F. nucleatum* exploits oxygen for energy conservation, as evidenced by its butyryl-CoA oxygen oxidoreductase system. Additionally, the ModRS two-component regulatory system plays a crucial role in orchestrating metabolic adaptations to counterbalance the inactivation of the oxygen poisoning of pyruvate formate lyase. These adaptive mechanisms likely support *F. nucleatum’s* survival in the oral biofilm and its dissemination to extraoral sites, contributing to its association with systemic diseases, including cancer. A deeper understanding of these oxygen adaptation strategies may inform novel therapeutic interventions aimed at mitigating the pathogenic potential of *F. nucleatum* in both oral and systemic infections.

## SUPPLEMENTAL MATERIAL

Table S1 jb.00090-25-s0001.docx Gene numbers for *F. nucleatum* subsp. *nucleatum* ATCC 25586 and ATCC 23726.

ASM does not own the copyrights to Supplemental Material that may be linked to, or accessed through, an article. The authors have granted ASM a non-exclusive, world-wide license to publish the Supplemental Material files. Please contact the corresponding author directly for reuse.
