# Supplementary material for: Fusobacterium nucleatum: strategies for adapting to aerobic stress
Source: J Bacteriol. 2025 Jun 6;207(7):e00090-25. doi: 10.1128/jb.00090-25 (PMC12288474; doi:10.1128/jb.00090-25)
Supplement: Table S1 — Gene numbers for F. nucleatum subsp. nucleatum ATCC 25586 and ATCC 23726. [file jb.00090-25-s0001.docx]

# **Supplemental Material**

# ***Fusobacterium nucleatum*: Strategies for Adapting to Aerobic Stress**

Alexandra K. McGregor^1^, Kirsten R. Wolthers^1^

^1^Department of Chemistry, University of British Columbia, Okanagan campus, 3247 University Way, Kelowna, Canada, V1V 1V7

Corresponding author: Kirsten Wolthers

email: kirsten.wolthers@ubc.ca

keywords: *Fusobacterium nucleatum*, aerobic stress, flavodiiron proteins, glycyl-radical enzymes, methionine sulfoxide reductase, ModRS.

| **Gene (Predicted Functions)** | **Strain 25586 Gene #** | **Strain 23726 Gene Number** |
| --- | --- | --- |
| *ahpC* (peroxiredoxin) | FN1983 | HMPREF0397_1235 |
| *ahpF* (peroxiredoxin reductase) | FN1984 | HMPREF0397_1236 |
| *msrAB1* (methionine sulfoxide reductase) | FN0188 | HMPREF0397_0774 |
| *msrAB2* (methionine sulfoxide reductase) | FN0803 | HMPREF0397_1367 |
| *trx* (thioredoxin) | FN0093 | HMPREF0397_1543 |
| *trxR* (thioredoxin reductase) | FN1163 | HMPREF0397_1095 |
| *rbr* (rubrerythrin) | FN0455 | HMPREF0397_1176 |
| *fdp* (flavodiiron protein) | FN1423 | HMPREF0397_1186 |
| *bcr* (butyryl-CoA reductase) | FN1424 | HMPREF0397_1187 |
| *fdp* (flavodiiron protein) | FN0512 | HMPREF0397_2092 |
| *bcd* (butyryl-CoA dehydrogenase) | FN0783 | HMPREF0397_1384 |
| *etfB* (electron transfer flavoprotein subunit B) | FN0784 | HMPREF0397_1383 |
| *etfA* (electron transfer flavoprotein subunit A) | FN0785 | HMPREF0397_1382 |
| *atoA* (acetoacetate:butyryl-CoA transferase α subunit) | FN0272 | HMPREF0397_1422 |
| *atoD* (acetoacetate:butyryl-CoA transferase β subunit) | FN0273 | HMPREF0397_1421 |
| *hbd* (3-hydroxybutyryl-CoA dehydrogenase) | FN1020 | HMPREF0397_0156 |
| *cro* (3-hydroxybutyryl-CoA dehydratase) | FN1019 | HMPREF0397_0155 |
| *hutU* (imidazolone-propionate hydrolase) | FN1401 | HMPREF0397_1209 |
| *hutI* (imidazolonepropionase) | FN1404 | HMPREF0397_1206 |
| *ftcd* (forminotetrahydrofolate cyclodeaminase) | FN1405 | HMPREF0397_1205 |
| *hutH* (histidine ammonia lyase) | FN1406 | HMPREF0397_1204 |
| *ftcd* (glutamate formiminotransferase) | FN1407 | HMPREF0397_1203 |
| *fhs* (formate tetrahydrofolate ligase) | FN2082 | HMPREF0397_1093 |
| *megL* (methionine γ-lyase) | FN1419 | HMPREF0397_1190 |
| *por* (pyruvate flavodoxin oxidoreductase) | FN1421 | HMPREF0397_1192 |
| *nrdD* (Class III RNR) | FN0311 | HMPREF0397_0118 |
| *nrdG* (Class III GRE-AE) | FN0312 | HMPREF0397_0117 |
| *nrdA* (Class I RNR α subunit) | FN0102 | HMPREF0397_1717 |
| *nrdB* (Class I RNR β subunit) | FN0103 | HMPREF0397_1716 |

**Table S1**. Select gene numbers for *F. nucleatum* subsp. *nucleatum* ATCC 25586 cross-listed with *F. nucleatum* subsp. *nucleatum* ATCC 23726
